# Optimization of Classification Strategies of Acetowhite Temporal Patterns towards Improving Diagnostic Performance of Colposcopy

**DOI:** 10.1155/2017/5989105

**Published:** 2017-07-04

**Authors:** Karina Gutiérrez-Fragoso, Héctor Gabriel Acosta-Mesa, Nicandro Cruz-Ramírez, Rodolfo Hernández-Jiménez

**Affiliations:** ^1^Biomedical Research Center, Universidad Veracruzana, Xalapa, VER, Mexico; ^2^Artificial Intelligence Research Center, Universidad Veracruzana, Xalapa, VER, Mexico; ^3^Obstetrician and Gynecologist, Private Practice, Xalapa, VER, Mexico

## Abstract

Efforts have been being made to improve the diagnostic performance of colposcopy, trying to help better diagnose cervical cancer, particularly in developing countries. However, improvements in a number of areas are still necessary, such as the time it takes to process the full digital image of the cervix, the performance of the computing systems used to identify different kinds of tissues, and biopsy sampling. In this paper, we explore three different, well-known automatic classification methods (*k*-Nearest Neighbors, Naïve Bayes, and C4.5), in addition to different data models that take full advantage of this information and improve the diagnostic performance of colposcopy based on acetowhite temporal patterns. Based on the ROC and PRC area scores, the* k*-Nearest Neighbors and discrete PLA representation performed better than other methods. The values of sensitivity, specificity, and accuracy reached using this method were 60% (95% CI 50–70), 79% (95% CI 71–86), and 70% (95% CI 60–80), respectively. The acetowhitening phenomenon is not exclusive to high-grade lesions, and we have found acetowhite temporal patterns of epithelial changes that are not precancerous lesions but that are similar to positive ones. These findings need to be considered when developing more robust computing systems in the future.

## 1. Introduction

Cervical cancer is a multifactorial disease that is the result of a series of alterations in the cervical epithelia, which lead to cancer when they are not treated early. In 2012, 528,000 new cases were diagnosed worldwide; 85% occurred in developing countries. In the same year, almost 9 out of every 10 women around the world who died of cervical cancer lived and died in low- to middle-income countries. For prevention programs to be effective, women with positive screening test results must receive effective treatment, so a “screen-and-treat” approach or a “screen, diagnose, and treat” approach is recommended [1].

There are different methods of diagnosing cervical cancer, including conventional cytology (Pap smear), liquid-based cytology (LBC), visual inspection with acetic acid (VIA), colposcopy, Human Papilloma Virus (HPV) DNA test, Hybrid Capture II, and a histopathological analysis. The diagnostic abilities of some of these tests depend on how they are implemented. The variability in cost as well as the need for infrastructure, equipment, and the training of human resources could be a determining factor for each one [2].

Patients with abnormal Pap smear findings are commonly evaluated by means of a colposcopy and directed biopsy. Colposcopy offers a direct visualization of the cervical epithelia and facilitates biopsies in order to establish a diagnosis by means of histopathological analysis [3–5]. Colposcopy includes the application of a 3–5% acetic acid solution to discriminate between normal and abnormal tissue. This reaction is called acetowhitening, and it produces a noticeable effect compared to the normal pinkish color of the surrounding epithelia found in the cervix [6]. The change of the optical properties of the cervical epithelia during this phenomenon has been studied, but it has not yet been fully understood [7–11].

The result of a colposcopy is based mainly on the acetowhitening reaction, but this reaction is not unique to precancerous lesions; it can also be found as a result of other situations, such as immature squamous metaplasia, congenital transformation zones, healing and regenerating epithelium (associated with inflammation processes), leukoplakia, and condyloma [6]. Therefore, the interpretation of a colposcopy is influenced by the experience of the specialist [12]. The sensitivity and specificity of traditional colposcopy vary depending on the conditions of each study, some of which have reported values of 83–98% and 48–66%, respectively [13, 14]. The main drawback of colposcopy is their lower level of specificity, which leads to a significant false positive rate. As such, acetowhite lesions associated with chronic cervicitis or squamous metaplasia can be confused with VPH infection or a precancerous lesion [15].

New methods to ensure a more accurate diagnosis are required, and several research projects have been undertaken, most of which have studied the problem based on modeling the acetowhitening reaction during a colposcopy using a temporal approach. Some of the preliminary studies included a reduced number of cases (6–38 patients) [16–23], while others considered different kinds of tissues, such as cervicitis, mature squamous epithelium, metaplasia, or colpitis [24–26]. However, there was variability in the acetowhite temporal patterns, possibly stemming from factors such as patient age, patient race, the stage of menstruation, or experimental conditions [26]. There is a need for standardizing the way in which colposcopy is performed, including a more uniform method of acetic acid application, improved viewing of the cervix, normalized lighting conditions, and accurate image registration, in order to improve diagnostic statistical accuracy [27].

Recently, some clinical trials conducted by a medical device manufacturer using a Dynamic Spectral Imaging (DSI) system have shown promising results [28–33]. However, there is also a study that argues the opposite and establishes that DySIS cannot replace conventional colposcopy with random biopsies [34].

The majority of clinical trials used the results of histopathological testing as their gold standard. Colposcopic impressions are confirmed by biopsies from all suspicious sites of precancerous lesions within the cervix, even though biopsy sampling is often a stressful and painful procedure for women, not to mention the problems it poses [24, 35]. However, taking more than one biopsy is a strategy to compensate for the limitations of colposcopic assessment. These findings highlight the need to improve both the sensitivity and specificity of colposcopy [36].

In most cases, DSI colposcopy has a higher sensitivity (82%) than conventional colposcopy with regard to distinguishing between normal and abnormal tissues, even when referral criteria are changed [31]. However, in order to distinguish low-grade lesions from high-grade lesions and cancer, long-term studies have reported that colposcopy has a sensitivity of approximately 56% [35]. Rarely do invasive carcinoma originate from smaller lesions, which would be harder to identify as suspicious through a colposcopy, but it is possible [37, 38]. These issues are challenging both traditional colposcopy and digital colposcopy. For example, some drawbacks of the DSI colposcope have been reported. During examination, it is recommended that the speculum be attached to the device, but this sometimes hinders the complete view of the transformation zone, especially in women with a retroverted uterus. DSI colposcopes also cause problems in other situations, such as when only part of the cervix can be visualized at one time or when there is an excess of blood or mucus in the cervix [30].

The main contribution of this paper is to evaluate the different models of acetowhite temporal patterns through a supervised learning approach in order to improve the diagnostic performance of digital colposcopy. We have found acetowhite temporal patterns of epithelial changes that are not precancerous lesions but that are similar to positive ones. These findings need to be considered when developing more robust computing systems in the future.

The analysis was carried out using some of the most representative methods of machine learning for the task of automatic classification: *k*-Nearest Neighbors (KNN), Naïve Bayes (NB), and C4.5. Firstly, experiments were carried out using only acetowhite temporal patterns based on binary classes. Additionally, an analysis using multivalued classes was carried out in order to identify the type of tissues that were misclassified.

The contents of this article are organized in five sections. In the next section, the materials and methods used in this research are described. The third section presents the results of the automatic classification methods, and one section focusing on a discussion of the findings is included. The fifth and final section presents ideas and conclusions.

## 2. Materials and Methods

### 2.1. Preparation of Subjects

This study encompassed 200 women. Given that some abnormalities were reported when analyzing their Pap smear tests, all of the patients were referred for a colposcopy. The average age was 34 (SD = 9); 88% claimed not to smoke; 54% reported having one sexual partner; the age average of first sexual intercourse was 18 years of age (SD = 4); 40% had Bilateral Tubal Occlusion (BTO) as a method of family planning; and only 2% used oral hormonal contraceptives.

Patients signed an informed consent form after the colposcopy procedure was explained to them. Said procedure was then performed. The speculum was introduced while the patient was in a gynecological position, and cotton swabs impregnated with saline solution were used to clean any cervical mucus. The appearance of cervical tissue was observed, and approximately three milliliters of 3% acetic acid solution was introduced into the cervical area. A cotton swab was placed in the lower part of the cervix to absorb the excess solution. In cases where a biopsy was obtained, Monsel's solution was used to stem the bleeding at the site from which the tissue sample was taken.

The colposcopy test allows us to observe if there are any changes in the appearance of the cervical epithelia. If these alterations lead to any suspicion of a lesion, a physician can take a tissue sample (biopsy) for histopathological analysis. Of all of the patients, a biopsy was obtained from 100 of them, while it was not necessary for the other patients as the physician did not find any changes that would suggest a lesion. Therefore, 93 cases tested positive (+) and 7 tested negative (−) for precancerous cervical lesions based on histopathological analysis and 100 tested negative (−) based on the colposcopy.

### 2.2. Data Acquisition

The data acquisition process was carried out using a tool based on MATLAB technical computing language (R2009a) using a Vasconcellos CP-M1225 colposcope with a STC-N63BJ camera. A green filter was used to acquire the images from the colposcope because a previous study reported higher values of sensitivity in the green component of the RGB color space [20].

The dimension of the images was 352 × 240 pixels and they were stored as separate files in BMP format. Before the application of acetic acid, 10 images were obtained as a point of reference. Then, 180 images were taken during a period of 3 minutes with a sampling frequency of 1 frame/second.

When the acquisition process was completed, the colposcopist selected a region where the biopsy was obtained on one of the previously acquired images. In cases where a biopsy was not necessary, a representative region of the tissue type was selected in the image. Based on our previous experience, image processing was carried out in grayscale [18].

### 2.3. Preprocessing

Slight movements attributed to nervousness, muscle tone, and breathing commonly occur during the image acquisition process. Therefore, a technique called registration was used to align the sequence of images and achieve anatomical correspondence. Basically, this process can be categorized based on their nature as area-based or feature-based methods. The area-based methods work directly with image intensity values. In contrast, the feature-based methods are based on the extraction of salient structures in the images [39].

Colposcopic images do not contain many differences throughout the sequence because, on a local basis, they differ only through translation, so a classical method in the category of area-based methods was applied. The representative area-based method is the normalized cross-correlation. Windows of predefined size from the input and reference images were used to calculate a similarity metric until its maximum value was reached. The input and reference images were updated starting with the first and second images of the sequence, respectively; then the input and the reference images were redefined by the second and the third images; and so on [40].

### 2.4. Time Series Extraction

**Figure 1:**
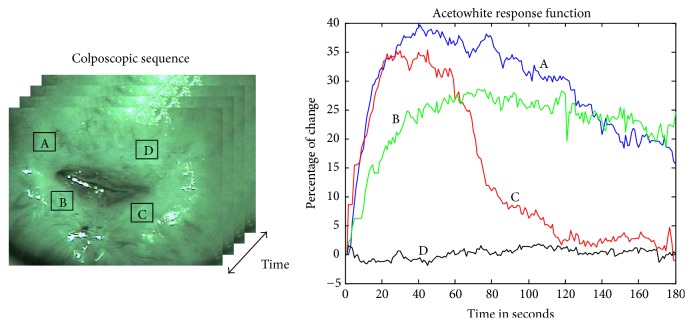
Acetowhite temporal patterns of different regions in a colposcopic sequence.

The colposcopic image sequence can be represented as a sequence of 2D images *I*(*x*, *y*) with acquisition time *t*. Therefore, there are (*x∗y*) pixels, and the intensity value *I* of each pixel over time is used to construct a time series of length* t* (acetowhite response function (Awrf)).  Figure 1 shows Awrfs of different regions in a colposcopic sequence that describes acetowhite temporal patterns of different kinds of epithelia.

As it was necessary to compare time series from different subjects, a standardization method was applied, which calculates the percentage of change of the signal with respect to the basal value. Once standardization was accomplished, data was represented by mean a polynomial model obtained experimentally by analyzing the behavior of the time series [17]. Three different representations of data were then used in this study: standardized data, data adjusted to the polynomial model, and data of the parameters of the model. These representations reduce the noise of raw data, and they were compared based on task classification. The first representation refers to standardized data by calculating the percentage of change and an example. The last two correspond to the following polynomial model:

(1) $$\begin{array}{c} \text{Awrf}=\theta_{0}+\theta_{1}t+\frac{\theta_{2}}{t}+\frac{\theta_{3}}{t^{2}}+\frac{\theta_{4}}{t^{3}}, \end{array}$$

where

  Awrf is acetowhite response function;
 
*θ* is explanatory variables;
 
*t* is time series.

**Figure 2:**
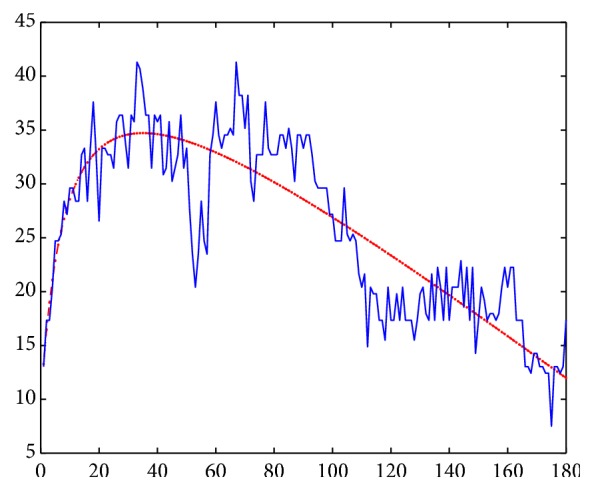
Example of standardized data and data adjusted to a polynomial model.

Figure 2 shows an example of data adjusted to the polynomial model on the dotted line, with the standardized representation being represented by the solid line. We can observe noise between the data adjusted to the polynomial model and the standardized representation as a result of changing lighting conditions during examination. These representations of the time series include continuous values; however, there are methods to compress data and facilitate their computational treatment. The process of mapping variables with continuous values into discrete ones is called discretization. It produces approximations of time series through discretization schemes on the *x*- and *y*-axes.

**Figure 3:**
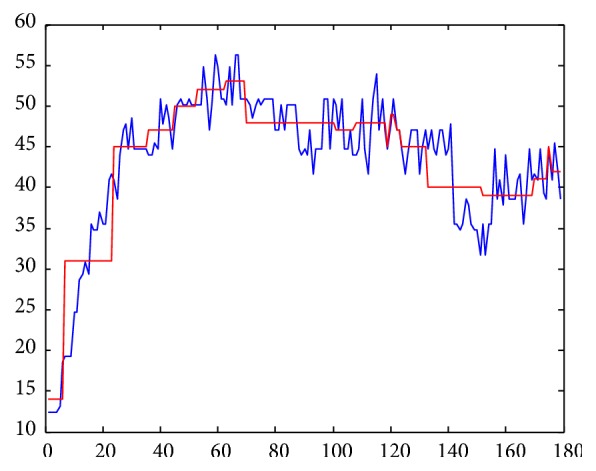
Discretized representation Piecewise Linear Approximation (PLA).

**Figure 4:**
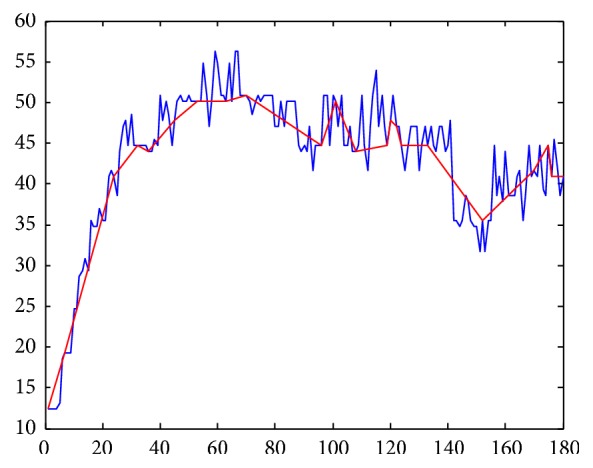
Discretized representation Piecewise Slope Approximation (PSA).

The reduction of dimensionality along the *x*-axis is obtained by dividing the total length of the time series into fragments of a certain size (word size). It is also necessary to establish a number of intervals along the *y*-axis in order to compress the values of the time series (alphabet size) [41]. In this study, we applied the algorithm proposed by Acosta-Mesa et al. [42] in order to discretize the time series, which optimizes the word and alphabet size as a single parameter by means of an evolutionary programming approach. The time series were divided according to the size of segments from the discretization model obtained by the algorithm, and a discrete value for each segment was calculated based on two criteria:

- Piecewise Linear Approximation (PLA): the average for each segment along the *x*-axis is calculated and is mapped to a discrete value to find the interval along the *y*-axis that includes it [43]. The segment size (word size) and the intervals (alphabet size) were established according to the discretization model shown in Figure 3.
- Piecewise Slope Approximation (PSA): this algorithm is similar to the previous one, but, in this case, the slope is calculated for each segment, and this value is then mapped among 7 possible values: 3 negative values, 3 positive values, and the number 0, representing no change.  Figure 4 shows an example of a time series represented by this method.

We decided to use different representations of time series, including continuous and discrete values, in order to observe which one would be better for the automatic classification task. This issue is fundamental in digital colposcopy because even though, in general, women found that the additional time the DSI colposcopy took was acceptable, some of them thought the time it took made them feel more uncomfortable. Furthermore, women ranked test accuracy as the most important characteristic, followed by rapid testing and comfort. Quick notification of results and costs were considered to be the least important characteristics [44]. Accordingly, the findings of this study try to contribute to the accuracy of colposcopy and faster testing to reduce the dimensionality of the data compiled.

We also wanted to compare our results, using discrete representations (PLA and PSA), with results obtained previously by Acosta-Mesa et al. [19]. Although both of the studies considered the discrete representations mentioned above, that one used fixed-size segments on word and alphabet parameters, but, in this study, the size of these parameters was variable, and it was established using the discretization model described in [42].

### 2.5. Time Series Databases

In this study, we used two databases. The first one included 200 cases with binary classes, covering one time series obtained from the colposcopic image sequence for each patient. That database encompassed 100 cases with a biopsy and 100 without a biopsy; however, in the subset of patients with a biopsy, 3 of the patients obtained a negative result for a cervical precancerous lesion. In the second database, the 200 cases were included, but there were multivalue classes. Based on the nature of the study and previous research into the dynamics of the acetowhitening phenomenon [17], the time series are referred to as acetowhite temporal patterns.

### 2.6. Supervised Learning

The aim of supervised learning is to predict a class label for a new example based on a model constructed from a set of training examples, where each one has its own corresponding class label. Hence, supervised learning algorithms try to correctly determine the class label for unseen circumstances. When a class label is categorical, learning is referred to as classification [45]. There are different models for the classification process, including* k*-Nearest Neighbors (KNN), Naïve Bayes (NB), and decision trees (ID3, C4.5, or J4.8), among others [46]. In this study, the KNN, NB, and C4.5 automatic classification methods have been employed, using their algorithms in the WEKA software (IBk, Naïve Bayes, and J4.8, resp.). These are described in the following subsections.

#### 2.6.1. *k*-Nearest Neighbors (KNN)

KNN is a type of instance-based learning method, and its output is a class membership. This means that a new example is assigned to the class most common among its *k* nearest neighbors from a training dataset. When the value of *k* is equal to 1, the class of the most similar observation within the training set is assigned. The observations in the dataset are the time series extracted from the colposcopic images. There are different measures of similarity, but the most common is Euclidean distance [45].

#### 2.6.2. Naïve Bayes (NB)

NB is a simple probabilistic classifier based on Bayes' theorem. This method can predict the probability that a new example belongs to a class given the observations contained in the training dataset. This method is useful because it provides a way to calculate these probabilities.

(2) $$\begin{array}{c} p\left(\;c_{j}\;|\;d\;\right)=\frac{p\left(d\;|\;c_{j}\right)p\left(c_{j}\right)}{p\left(d\right)}, \end{array}$$

where

 
*p*(*c*_*j*_∣*d*) is probability of class *c*_*j*_ given the observation *d*;
 
*p*(*d*∣*c*_*j*_) is probability of the observation *d* given class *c*_*j*_;
 
*p*(*c*_*j*_) is probability of occurrence of class *c*_*j*_;
 
*p*(*d*) is probability of occurrence of the observation *d*.

#### 2.6.3. C4.5

The decision tree is a structure resembling a flow chart, similar to that of a tree, where each internal node denotes the testing of an attribute. In this study, each discrete value of the time series was considered as an attribute. The decision tree has branches, with each branch representing a departure from the test and each terminal node (“leaf node”) representing a class label. The node at the top of the tree is the root node [47]. The paths from the root to the leaf represent classifications rules.

The performance of these three well-known classification methods was assessed using continuous and discrete values. On a more specific level, the methods included different representations of continuous values: standardized data, data adjusted to a polynomial model, and data of the parameters of the model.

### 2.7. Evaluation Methods

The most widely used basic measures to evaluate the performance of automatic classification methods are accuracy, sensitivity, and specificity. Sensitivity is equivalent to true positive rate (TP rate) and recall; specificity is equivalent to 1—false positive rate (FP rate). Another measure is precision, and the precision-recall curve is based on this. Matthews Correlation Coefficient (MCC) and* F*-Measure are also useful, but they are less frequently used. MMC is a correlation coefficient calculated from all four values from the confusion matrix. The* F*-Measure score is a harmonic mean of recall and precision. ROC and PRC area under curve (AUC) scores are useful when comparing the performances of multiple classifiers [48–50]. All these measures and the confusion matrix shown in Table 1 stem from the reporting of the WEKA results.

In this study, class = 1 represents a positive case of cervical precancerous lesion, while class = 0 corresponds to a negative one. The confusion matrix summarizes the number of test cases correctly (*f*_11_ + *f*_00_) and incorrectly (*f*_10_ + *f*_01_) predicted by the method. Accordingly, *f*_11_ represents the true positive cases (TP), *f*_10_ represents the false negative cases (FN), *f*_01_ represents the false positive cases (FP), and *f*_00_ represents the true negative cases (TN).  Table 2 shows some of the basic measures derived from confusion matrix.

In order to compare the performance of the learning algorithms from a statistical point of view, a cross-validation method was applied, consisting of dividing the data into two sets: training and validation. In the basic form of cross-validation, the database is divided into* k*-partitions (*k*-fold cross-validation,* k*-fold CV) of equal or almost equal size. The procedure comprises making *k* iterations of training and empirical validation executed consecutively. Thus, a different partition of data is maintained for empirical validation while the remaining *k* − 1 partitions are used for learning each iteration [51].

## 3. Results

In this section, an analysis regarding different models of acetowhite temporal patterns by means of supervised learning approach is shown. Experiments included three machine-learning methods enabled in WEKA in order to compare their performance using temporal data on a database encompassing 200 cases. The information is organized into two subsections. The first one shows results of experiments performed considering a binary class of the acetowhite temporal patterns. This means that one acetowhite temporal pattern was included from each patient, which was labeled based on colposcopic or histopathological testing. The analysis was carried out by trying different data representation methods: standardized data, data adjusted to polynomial model, and data of the parameters of the model. In this way, the capacity of those representation methods to ensure the relevance of the data of the classification tasks was also evaluated. The second subsection shows an analysis carried out with multivalue classes including the six original labels. The analysis of second subsection was applied to the automatic classification method with the best ROC and PRC AUC scores obtained in binary classification.

### 3.1. Binary Classes Classification

The class assigned to each case in these experiments was binary: positive or negative for a cervical precancerous lesion.  Table 3 shows the results of weighted average performance obtained from detailed accuracy by class of WEKA for the different methods and models. According to the process of time series extraction described above (see Section 2.4), when data was represented by a discretized model using PLA and PSA methods, variable size segments were established.

According to results shown in Table 3, the highest ROC and PRC AUC scores were obtained by the KNN method and discrete PLA representation of acetowhite temporal patterns.  Table 4 shows its confusion matrix.

The evaluation measures described in Table 2 were calculated for the confusion matrix shown in Table 4. The values of sensitivity, specificity, and accuracy reached using the KNN method and discrete PLA representation were 60% (95% CI 50–70), 79% (95% CI 71–86), and 70% (95% CI 60–80), respectively.

### 3.2. Multivalue Classification of Classes

In order to better explain the classification process, an analysis at subclass level using the KNN method and discrete PLA representation is shown here. Before the binarization of classes, six labels describing different types of cervical tissue were considered in this study: (1) atrophy, (2) inflammation, (3) ectopy, (4) normal, (5) low-grade squamous intraepithelial lesion (LSIL), and (6) high-grade squamous intraepithelial lesion (HSIL).  Table 5 shows how the classes and subclasses are matched. Subsequently, in this subsection, the subclasses are referred to as simply classes.

Table 6 shows the confusion matrix for multivalue classification of classes using the KNN method and discrete PLA representation. Detailed accuracy by class of this method is shown in Table 7.

It can be observed in Table 7 that “Normal” and “HSIL” classes obtained the highest ROC and PRC AUC scores. The values of sensitivity, specificity, and accuracy achieved for the “Normal” class by means the KNN method and discrete PLA representation through multivalue classification of classes were 92%, 49%, and 60%, respectively. Besides, values of these measures for the “HSIL” class were 71%, 73%, and 73%, respectively.

**Figure 5:**
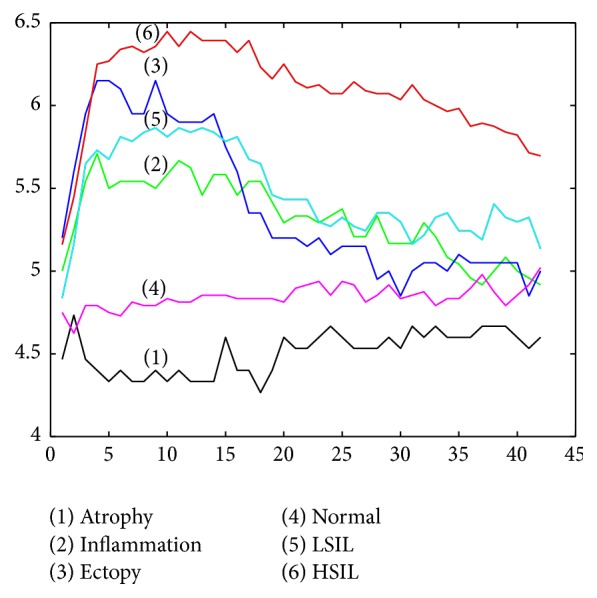
Acetowhite temporal patterns of different kind of epithelia based on mean of data using discrete PLA representation.

In order to explore graphically the acetowhite temporal patterns of different kind of epithelia, the mean of data for each class using discrete PLA representation is shown in Figure 5.

## 4. Discussion

The experiments presented in this study have shown that the automatic classification methods using a temporal approach can discriminate between a certain percentage of normal and abnormal cases. The technical measures included in this work allowed us to evaluate the performance of the automatic classification methods. The system of relationships among them and its generalization to multiclass contingency tables have been reported [52]. According to results of binary classification, the KNN method and discrete PLA representation showed better performance than the other methods evaluated in this study. Our results suggest that a discretized representation could make it possible to achieve lower computational cost and at the same time preserve relevant information to classification task. However, the highest values of the metrics are not optimal. This could be related to some factors that have a potential effect on accuracy like the prevalence of disease, verification bias when no biopsies are taken from colposcopically negative sites on the cervix, inclusion of LSIL in the definition of abnormal, and whether the colposcopic impression or the biopsy is used as the end point [32].

Additionally, weighted average results of multivalue classification of classes using the KNN method and discrete PLA representation showed a general poor performance. Nonetheless, it was observed that classes with more cases (Normal and HSIL) obtained better performance by class. This issue could be attributed to unbalanced classes on data [48].

On the other hand, we have found graphic similarity between acetowhite temporal patterns of cervical tissues considering normal (ectopy and inflammation) and abnormal cases (LSIL and HSIL). This could be explained as acetowhitening is not exclusive to cervical precancerous lesions, as it is also seen in other situations, such as immature squamous metaplasia, congenital transformation zones, healing and regenerating epithelia (associated with inflammation processes), leukoplakia, and condyloma [6]. In addition to effect on performance of automatic classification methods, these findings should be considered for developing digital colposcopy systems able to discriminate among different kinds of cervical epithelia [32].

## 5. Conclusions

The temporal approach applied in this study quantitatively represented the dynamics of the acetowhitening phenomenon in order to discriminate, with a certain degree of accuracy, between normal and abnormal cases. Furthermore, it was shown that the highest metric values were obtained using PLA discretized representation. This suggests that this kind of representation leads to a lower computational cost and less time required to process a complete image.

However, it is necessary to continue working to improve the automatic classification methods in order to decrease cases of false negatives and false positives. Identifying the former is important because it could determine if cervical cancerous lesions are diagnosed in earlier stages and subsequently the proper treatment could be commenced. In cases of false positives, unnecessary and possibly invasive procedures could be avoided. Finally, all efforts to develop better methods of diagnosing cervical cancer can contribute to the health of women who are at risk of this disease.

## Figures and Tables

**Table 1:** Confusion matrix.

		Predicted	
		Class = 1	Class = 0
Actual	Class = 1	*f*_11_	*f*_10_
	Class = 0	*f*_01_	*f*_00_

**Table 2:** Evaluation measures from the confusion matrix.

Measure	Formula
Accuracy	(TP + TN)/(TP + TN + FN + FP)
Sensitivity	TP/(TP + FN)
Specificity	TN/(TN + FP)

**Table 3:** Performance of automatic classification methods using acetowhite temporal patterns on a dataset of 200 cases.

Method	Model	Classified instances (%)		TP rate	FP rate	Precision	Recall	*F*-Measure	MCC	ROC area	PRC area
		Correctly	Incorrectly								
IBk	Standardized	70	30	0.700	0.309	0.700	0.700	0.699	0.395	0.721	0.683
	Adjusted	69	31	0.690	0.319	0.689	0.690	0.689	0.375	0.717	0.679
	Parameters	64	36	0.635	0.375	0.634	0.635	0.633	0.263	0.632	0.599
	PLA	70	30	0.700	0.313	0.701	0.700	0.697	0.395	0.732	0.713
	PSA	62	38	0.620	0.437	0.778	0.620	0.539	0.327	0.610	0.608

Naïve Bayes	Standardized	69	31	0.690	0.329	0.695	0.690	0.684	0.377	0.713	0.681
	Adjusted	69	31	0.685	0.333	0.689	0.685	0.679	0.366	0.708	0.673
	Parameters	53	47	0.525	0.459	0.537	0.525	0.520	0.067	0.540	0.543
	PLA	65	35	0.645	0.385	0.658	0.645	0.627	0.289	0.697	0.669
	PSA	61	39	0.605	0.409	0.603	0.605	0.601	0.200	0.624	0.617

C4.5	Standardized	68	32	0.675	0.330	0.674	0.675	0.675	0.346	0.627	0.592
	Adjusted	64	36	0.640	0.361	0.641	0.640	0.640	0.279	0.618	0.582
	Parameters	55	45	0.545	0.471	0.541	0.545	0.539	0.076	0.526	0.520
	PLA	65	35	0.645	0.361	0.644	0.645	0.645	0.285	0.652	0.611
	PSA	64	36	0.635	0.379	0.634	0.635	0.631	0.262	0.643	0.611

**Table 4:** Confusion matrix for the KNN method and discrete PLA representation.

Classified as →		a	b
Positive	a	56	37
Negative	b	23	84

**Table 5:** Classes and subclasses.

Class	Subclass	Cases	Biopsy	No biopsy
Negative	(1) Atrophy	15	0	15
	(2) Inflammation	24	6	18
	(3) Ectopy	20	0	20
	(4) Normal	48	1	47

Positive	(5) Low-grade squamous intraepithelial lesion (LSIL)	37	37	—
	(6) High-grade squamous intraepithelial lesion (HSIL)	56	56	—

*Total*		200	100	100

**Table 6:** Confusion matrix for multivalue classification of classes.

Classifies as →	a	b	c	d	e	f
a = atrophy	0	0	0	14	0	1
b = inflammation	0	0	0	13	1	10
c = ectopy	0	0	0	12	0	8
d = normal	0	0	0	44	0	4
e = LSIL	0	0	0	22	0	15
f = HSIL	0	0	0	16	0	40

**Table 7:** Performance of the KNN method and discrete PLA representation using multivalue classification of classes on a dataset of 200 cases.

Class	Classified instances (%)		TP rate	FP rate	Precision	Recall	*F*-Measure	MCC	ROC area	PRC area
	Correctly	Incorrectly								
Atrophy	0	100	0.000	0.000	0.000	0.000	0.000	0.000	0.626	0.108
Inflammation	0	100	0.000	0.000	0.000	0.000	0.000	0.000	0.582	0.148
Ectopy	0	100	0.000	0.000	0.000	0.000	0.000	0.000	0.736	0.225
Normal	92	8	0.917	0.507	0.364	0.917	0.521	0.358	0.784	0.490
LSIL	0	100	0.000	0.006	0.000	0.000	0.000	−0.034	0.494	0.178
HSIL	71	29	0.714	0.264	0.513	0.714	0.597	0.415	0.738	0.448
Weighted Average	42	58	0.420	0.197	0.231	0.420	0.292	0.196	0.677	0.324
